# From across the Seas

**Published:** 1916-07-01

**Authors:** 


### FROM ACROSS THE SEAS.

### Making the Blind to See.

THE German journal *Deutsche Optische Wochenschrift* recently contained an article by Professor L. Zehender, a Berlin ophthalmic surgeon, describing some experiments in relation to an apparatus for use as a "substitute for sight." From the country which so recently has produced simulated meat and other counterfeit substances, it is not surprising to receive tidings of simulated sight for the blind. But the news does not appear to have had much effect on our English Press; one well-known daily notices the article under the heading "From German Sources," but it is to the *Braille Review* that we owe the following description of an apparatus which is not too easy to describe, and of the manner in which it has been introduced to the world:—

Everyone knows that the sun may be felt as well as seen, and probably not a few schoolboys have experienced the torment of being subjected to the effects of the focussing of a "burning-glass" upon their skins. Yet this is made the basis of a lengthy disquisition on a substitute for sight. It is proposed with every appearance of seriousness that a burning-glass should be placed in such a relation to the breast of the blind subject that the light focussed by the lens should fall accurately upon the skin: means would be taken by the interposition of screens to prevent burning, and by a process of training the subject would learn to see by means of the feel of the warm picture on his chest. The educational process would be carried out by placing a series of stencils on the chest which would leave slits, circles, and later on letters and signs exposed to the heat rays, and the sense of heat perception would be increased by its cultivation.

### Probationers and War Weddings.

WE learn from the *South African Nursing Record* that the question of a nurse taking advantage of leave of absence to engage in a war-wedding is a problem arising in our Colonial hospitals as well as at Home. On this point our contemporary reports that an interesting and unusual discussion arose at the April meeting of the Johannesburg Hospital Board. It was reported by the matron that a probationer had, during her leave, got married, and the medical superintendent recommended, in accordance with the hospital regulations, that the probationer should be discharged. The direction the discussion took was that this was a war wedding, that the bridegroom had immediately gone to the Front, and that under the exceptional circumstances the breach of the regulations might be overlooked. The hospital has taken no action at present in the matter, which has been referred to the house committee. The *Record*, in reviewing the general question, is of opinion that a woman cannot do two things at once, any more than a man, and marriage is the greatest and most important of all the experiments she can undertake. With very few exceptions, the nurse who takes up matrimony must give up nursing. Both of them demand a complete concentration of thought and energy, and they are not, as a rule, compatible.

